# Suspension Fertilizers Based on Waste Phosphates from the Production of Polyols

**DOI:** 10.3390/molecules27227916

**Published:** 2022-11-16

**Authors:** Paulina Bogusz, Piotr Rusek, Marzena Sylwia Brodowska

**Affiliations:** 1 Fertilizers Research Group, Łukasiewicz Research Network–New Chemical Syntheses Institute, Al. Tysiąclecia Państwa Polskiego 13a, 24-110 Pulawy, Poland; 2 Department of Agricultural and Environmental Chemistry, University of Life Sciences in Lublin, Akademicka 13, 20-950 Lublin, Poland

**Keywords:** phosphorus, critical raw materials, circular economy, waste management, liquid waste

## Abstract

Phosphorus raw materials are non-renewable, and their resources are shrinking faster and faster as a result of increased fertilizer production. This is due to the increasing population and the need to produce more food. Phosphorus, on the other hand, is one of the main nutrients of plants, without which it is impossible to conduct intensive agricultural production. There are no economically significant phosphate resources in Europe, so they must be imported. That is why it is so important to reduce losses and recover this element from waste streams, which was reflected in the new EU Regulation 2019/1009. A prospective option is to use waste phosphates from the production of polyether polyols. Previous studies show that they contain about 20% phosphorus. Due to their high water content, the most advantageous form of their application is the production of fertilizers in the form of a suspension. The aim of the study is to assess the possibility of using waste phosphates from the production of polyols as raw materials for the production of suspension fertilizers.

## 1. Introduction

Suspension fertilizers on an industrial scale were introduced in the United States in the late 1960s [1]. Although they are an American specialty, they have many followers in Canada, developed countries, Europe, and Asia [2,3]. The economics of production and the efficiency of using this form of fertilizer have contributed to a steady increase in their agricultural use starting from the 1970s [3].

In the United States, where these fertilizers are the most popular, their production takes place at satellite liquid fertilizer stations and base suspensions at fertilizer factories. The share of suspension fertilizers there accounts for about 1/3 of the entire liquid fertilizer market [4]. About 70% of suspension fertilizer mixtures are made of base suspensions and nitrogen solutions, while the remainder is produced on the basis of solid fertilizers [1].

The first fertilizer suspensions were developed by the Tennessee Valley Authority (TVA), which, in the late 1960s, improved the manufacture, handling, and use of stable suspensions [4]. Further research in the 1970s on optional raw materials, suspending agents, and processing techniques resulted in a spectacular improvement in product quality and versatility, thus ensuring commercial success, not only in the United States, but also beyond [5].

In the early 1980s, fluid fertilizer foundation was established to sponsor field work to determine the optimal use of liquid fertilizer systems in plant production [6]. Currently, liquid products, including suspensions, are gaining more and more popularity in the most developed markets. In terms of the global nitrogen market, liquid fertilizers currently account for around 12% of the total market [5,7].

Suspension fertilizer manufacturers offer a diverse range of products based on various techniques. The proposed production procedures are aimed at ensuring better stability of the suspensions, increasing the concentration of fertilizing components, and improving the bioavailability of the components.

A common practice in the production of suspension fertilizers is the production of base fertilizers that can be stored for a longer period, provided that they are periodically mixed. Depending on the demand, the base suspensions are enriched with appropriate raw materials to obtain a multi-component fertilizer with the desired composition, which requires application within several hours from the end of production [8,9].

Clear solutions of urea ammonium nitrate can be prepared for a nitrogen concentration of about 32% at normal use temperatures. The concentration of liquid suspension type fertilizers may exceed this limit, and processes for this have been developed in the United States. The hot 87% urea solution and the hot 88% ammonium nitrate solution are mixed and then cooled to 55 °C before being transferred to the gelling tank. An urea-to-ammonium nitrate ratio is used, which provides 70% of total urea nitrogen. Resultingly, it is the urea that crystallizes on subsequent cooling. Dry attapulgite clay is added to the solution in an amount constituting 1.5% of the product. The clay gelation tank is a baffled vessel that provides good mixing, equipped with recirculation by a centrifugal pump to assist the gelation. A corrosion inhibitor is added, if necessary. After this step, the mixture must be cooled rapidly below 45 °C in order to induce intense nucleation of small urea crystals. They are easy to hang and provide a large surface area for further crystal growth that may occur during storage. It is important to avoid the slow growth of large crystals that could block the operation of the equipment. Rapid cooling is achieved by passing the mixture through a forced draft spray tower. The partially cooled slurry is recirculated from the pond at the base of the tower. Crystal size can be controlled by varying the recycle rate (which is typically 15:1 from the product removal rate). The suspension prepared in this way and cooled is transferred to the warehouse [5,10].

The Luxen method for the production of suspension fertilizers is based on the use of ground raw materials with particles above 20 mesh, appropriate mixing speeds (1280 rpm for the clear solution mixing stage and 2620 rpm for the suspension mixing stage), maintaining the temperature in the range of 30–40 °C, and using xanthan gum as a stabilizing factor in the amount of 2.5% [11].

This method was used for the production of NPK suspension fertilizer with a composition of 25-7-7. The process consists in introducing a part (about 85%) of the ground urea, water, and the stabilizing agent—xanthan gum in the stage of mixing at a lower speed. The remainder of the urea, water, KCl, and MAP are added in a second mixing step with increased agitator speed and are mixed until homogenization is achieved. The urea added in the first mixing step dissolves completely to give a saturated solution with a three-dimensional network of xanthan gum. In the second stage of mixing, the remaining raw materials are introduced. The undissolved ingredients are kept in suspension in dispersion by the xanthan gum by Van der Wals forces, and the saturated solution gradually becomes supersaturated. The dispersed phase of the slurry consisted of urea crystals 1 added in the first mixing step, urea particles 2 added in the second mixing step, and KCl and MAP added in the third mixing step. The environment of the urea 1 particles by the xanthan gum particles prevented the formation of clusters, which limited the formation of large crystals during the cooling of the suspension [11].

The method of producing a MAP-based suspension fertilizer with the addition of fluoride ions developed by TVA consists in adding appropriate amounts of MAP, water, H_2_SiF_6_, and ammonia to the reactor in an amount ensuring a pH of about 6.5. Then, attapulgite clay was added in an amount of 0.75–1.5%. The total mixing time of all ingredients was 20 min. At the stage of ammonization, the temperature of the suspension was in the range of 65–93 °C and it was higher with the addition of fluorine [12].

The use of a nucleating agent in the suspension causes the excess fertilizer salts to crystallize in the form of fine crystals, which remain suspended in a saturated solution of the same salts [13,14]. Without the use of a nucleating agent, the crystals expand, settle to the bottom, and form a hard mass that makes the resulting product unusable [13].

According to the invention in US Patent 3,113,858, the beneficial effect of the nucleating agent can be improved by the unique combination of appropriate size selection of the solids and the cooling of the hot fertilizer solution prior to the addition of the solids. Essential requirements are that the hot fertilizer solution is cooled prior to the introduction of the fine particle solids. Pre-cooling minimizes the dissolution of the solids and, thus, reduces the subsequent recrystallization in the form of large crystals. The fine size promotes the dispersion of undissolved solid particles. The combination of these effects with the beneficial effects of the nucleating agent results in a fertilizer suspension with improved properties [13].

The order in which the materials are added is not critical as long as the necessary sequence of cooling the produced fertilizer solution and adding solids is followed. It may be desirable to cool the fertilising solution prior to adding the remaining liquid materials in order to obtain a more efficient use of the coolant used, but this is not necessarily the case [13].

It is essential that the temperature of the slurry quickly reaches ambient levels after the addition of solids. The inevitable partial dissolution of the solids gives some cooling, as does the addition of any make-up liquids. Therefore, it is necessary to cool the solution to at least such an extent that the further cooling by additional fluids and by dissolving the solid brings the temperature down to ambient temperature [13].

The initial comminution of the solids is important since the particle size of the solids in the final suspension depends in this case more on the initial size than on the degree of growth during recrystallization [13].

According to US patent 3,109,729, the best suspension is obtained when the amount of water is limited to the extent that at least 1/3 of the total nutrients are in the dispersed phase [14].

The nucleating agent is best added at a time when the salts start to crystallize out of solution. Then, it can fully cause rapid nucleation of crystallizing salts, resulting in the production of a large amount of very small easily suspended crystals and not a small amount of large crystals. However, the nucleating agent will have a beneficial effect, even if added after excess salt has crystallized out. When the ambient temperature changes, the salts alternately dissolve and recrystallize. The presence of a nucleating agent then prevents the formation of hard masses by recrystallizing salts, which would cause deposition and segregation of nutrients and interfere with the application [14].

When the slurry is prepared by the ammonification of phosphoric acid to obtain ammonium phosphate, it is desirable that all the salts in the solution be introduced at one stage of the process so that the nucleating agent can exert its full effect. This is most easily accomplished by adding salt prior to the ammonification of the acid so that the heat of the neutralization reaction raises the temperature of the mixture high enough to dissolve the salts. However, salts can also be added during or even after acid neutralization, if added immediately before cooling the solution. Adding salt prior to neutralization has the advantage that more of the heat of reaction goes to providing the heat to dissolve the salt rather than evaporating the water. If these steps are not implemented in production, excess salt may crystallize as a heavy mass, settling on the bottom of or clinging to the walls of the container, rendering the product unusable [14].

The nucleating agent can be one of several fine inert materials. Examples are clay, dolomite, and other naturally occurring materials [14].

A popular option is the slow-acting urea-formaldehyde suspension fertilizers produced by the polymerization of urea and formaldehyde. According to US patent 4,526,606, a good quality urea-formaldehyde suspension fertilizer can be obtained, which additionally, leaves no color. For some commercial applications, such as fertilizing lawns, the trait of color is quite important. Coloring refers to the tendency of dried fertilizer suspension particles to remain on a substrate for extended periods, e.g., grass blades, driveways, sidewalks, etc. The dried suspension appears as a white unsightly deposit. Coloring is inversely proportional to particle size, e.g., smaller particles color more than larger particles. Therefore, particle size considerations for a stable fertilizer suspension run counter to the non-staining needs [15].

The addition of a low-foaming surfactant promotes the dispersion of the fertilizer after application, helping to re-wet the dried water-insoluble fertilizer material. In other words, the surfactant helps to reduce the tendency to stain from fertilizer suspensions [15].

US patent 3,155,489 discloses a fertilizer slurry preparation method that uses nitrogen oxides to acidify the slurry of calcium compounds, thereby obtaining nitrogen without the need for nitric acid. The process according to the invention combines the steps of oxidation, absorption, and acidification and allows the use of low-grade phosphates and a cheap source of nitrogen [16].

Usually, nitric acid is used to acidify phosphate rocks. However, it is expensive and produces a by-product, calcium nitrate. Since calcium nitrate is a very hygroscopic substance, it creates problems in the production of solid fertilizers. In order to overcome these difficulties, the invention uses nitrogen oxides and produces the end product in the form of a liquid slurry. The cost advantage of the invention is largely due to the direct absorption of nitrogen oxides by aqueous suspensions of calcium compounds to form a fertilizer suspension of solid particles in a liquid medium [16].

US patent 4,133,670 describes a method for the production of a monoammonium phosphate (MAP) suspension fertilizer with improved storage parameters [17].

A troublesome problem with ammonium phosphate suspension fertilizers is the formation of relatively large crystals (20 mesh or more). These crystals reduce storage time and clog the spray nozzles and other critical points in the spray device. This, in turn, means that frequent stoppages and cleaning work are necessary during use [17].

The process of the invention comprises initially mixing a portion of the amount of monoammonium phosphate (MAP) with water and adding ammonia thereto to induce an immediate ammonia reaction. The remainder of the MAP is then added to make the final fertilizer composition. This division of the MAP additive (preferably 70–30%) serves to minimize the formation of large ammonium phosphate crystals in the fertilizer, thanks to the improved storage properties, and the fertilizer can be sprayed without fear of frequent clogging of the spray equipment [17].

Patent US 3,677,736 describes a method of obtaining a suspension fertilizer containing large amounts of urea, the solubility of which is quite low, which excludes the formation of highly concentrated solutions on its basis [18].

The urea formaldehyde liquid suspension has the advantage that one application will provide a dose of rapidly available nutrients along with a slowly available nitrogen reserve. Moreover, the suspended particles of urea, when applied to soil or lawn, leave a visible bloom, which can serve as an indicator of which parts of the area have been sprayed with the liquid fertilizer mixture and which still require fertilizer application [18].

The pH of the starting material should be higher than 7 to prevent premature formation of urea formaldehyde or other products resulting from side reactions. When the pH drops below 5, the rate of the reaction between urea and formaldehyde to form urea formaldehyde is increased. However, when the pH drops below 1, the acid destroys the urea before combining it with the formaldehyde. The urea-formaldehyde compounds obtained in this case are, moreover, highly insoluble and undesirable [18].

The starting material is preferably heated for 15 min at 40–70 °C. The reaction speed is very slow at temperatures below 30 °C. At temperatures close to 80 °C, highly insoluble compounds were formed [18]. 

It is important to mix, during the reaction, to obtain a finely divided urea formaldehyde product. If the mixing is insufficient, highly insoluble nitrogen compounds are formed. High shear mixing using a baffled turbine mixer is preferred when the reaction of urea formaldehyde is carried out at a reaction temperature below 60 °C. On the other hand, mixing with a pump or impeller type agitator gives satisfactory results when the reaction is carried out at a temperature above approx. 60 °C [18].

At the end of the reaction time, an alkaline material is added to the mixture to raise the pH above 5 and thereby complete the urea formation reaction. The pH of the product should not exceed 8, as it may cause the suspension to react with the container and introduce impurities into the mixture. In addition, the increase in alkalinity attacks the urea in the product suspension. Typically, supplemental plant nutrients are added after the urea-formaldehyde acid reaction to form the urea formaldehyde suspension due to better control of pH conditions [18].

Detailed characteristics of suspension fertilizers are presented in the review article: Suspension Fertilizers: How to Reconcile Sustainable Fertilization and Environmental Protection [19].

The paper presents the results of tests on the production of suspension fertilizers based on waste phosphate from the production of polyols. The composition of the tested fertilizers was selected for maize grown for silage, with the intention of checking their effectiveness in field experiments for this plant. The six proposed fertilizers differ in their phosphorus content and the addition of secondary fertilizing components and microelements.

In the stabilization of samples of suspension fertilizers, the performance of four different bentonites was compared. In the first step, their swelling capacity was tested, producing a 12% aqueous solution. Bentonites showing a high swelling factor were tested in the stabilization of the proposed tests of suspension fertilizers by examining their basic operational parameters: stability, density, viscosity, and fluidity.

## 2. Materials and Methods

### 2.1. Fertilizer Raw Materials

Fertilizers based on waste phosphates presented in the paper are to be a replacement for fertilizers based on monoammonium phosphate (MAP). Therefore, the tests used typical fertilizer raw materials most often used in the production of suspension fertilizers, with the exception of the raw material, which is a source of phosphorus.

In the research, waste phosphates from the production of polyols were used as the phosphorus raw material, the detailed characteristics of which are presented in this paper: The Possibility of Using Waste Phosphates from the Production of Polyols for Fertilizing Purposes [20].

Table 1, Table 2, Table 3, Table 4, Table 5 and Table 6 show the parameters of the remaining fertilizer raw materials used in the presented trials of suspension fertilizers.

### 2.2. Stabilizing Agent

For the stabilization of fertilizer suspensions, bentonites are the most frequently used raw material due to their price and availability. Four bentonites from Zakłady Górniczo-Metalowe “ZĘBIEC” in Zębcu S.A. were tested in the research. 

These are the bentonites selected for research:

Bentonite GM—due to the high content of montmorillonite, it has a high viscosity and perfectly binds materials. It is characterized by high water absorption—bentonite swells quickly.

Bentonite Special—a bentonite enriched with sodium cation (i.e., activated), which significantly improves its functional properties. It increases the swelling ratio, i.e., bentonite hydration.

Bentonite Special 45—high-quality bentonite with a high montmorillonite content is used for its production. The suspensions made of Special 45 bentonite have excellent rheological and thixotropic properties, which translates into very good stabilization. Thanks to the high thixotropy, they keep the soil grains suspended.

Bentonite SN—is universally applicable and works well in many industries. It is used as an additive to animal feed, in the production of concrete, plasters, paints and varnishes, in the food and pharmacological industries, and in the sewage industry for wastewater treatment.

GM, Special and Special 45 bentonites with 75% montmorillonite content were activated with sodium cations. And SN bentonite is only ground and dried calcium bentonite that is not subject to the activation process.

### 2.3. Composition of Fertilizers

Two series of fertilizers were prepared. In the first series, three fertilizers were proposed with the percentage of the main NPK nutrients: 9.5-4-11 (4 × 2.4: 1: 2.8). Fertilizer 1 contains only the main nutrients (N, P, K), fertilizer 2 is additionally enriched with secondary nutrients (Mg, S), and fertilizer 3 is enriched with microelements (Table 7).

In the second series, the phosphorus dose was increased by 2% with the same content of nitrogen, potassium, secondary nutrients, and microelements as in the fertilizers in the first series. The composition of series II fertilizers (fertilizer 4, 5 and 6) is presented in Table 8.

The proposals of six suspension fertilizers were selected so as to easily compare the yield-generating efficiency of maize in relation to the amount of phosphorus component and depending on the addition of secondary nutrients and microelements.

### 2.4. Research Methodology

#### 2.4.1. Production of Suspension Fertilizers

Suspension fertilizers were produced using a high-speed DISPERMAT mixer with a dissolver type mixer. Appropriate amounts of liquid raw materials were metered into the reactor and mixed at the speed of 800 rpm for about 5 min. Then, solid raw materials were gradually dosed, increasing the stirrer speed to 1500 rpm. After the dosing of all raw materials was completed, the suspension was mixed for about 15 min.

#### 2.4.2. Stability Study

The basic study determining the durability of the prepared suspensions is a sedimentation test conducted for 48 h in order to observe all the phenomena occurring in the suspension from its production to application or complete degradation. 48 h is considered a satisfactory time to keep the suspension in a stable form. This is the minimum time that allows the suspension to be transported and applied. In order to measure the stability, the tested suspensions were placed immediately after production in measuring cylinders with a stopper of a capacity of 100 mL and a diameter of 30 mm, and the changes taking place were observed by measuring the height of delamination.

#### 2.4.3. Castability Testing

The pourability test was performed at the temperature of 20 °C with the use of a 100 mL Ford cup with a 4 mm diameter discharge nozzle. After the cup was fully filled with the tested suspension, the time of free flow of all the liquid through the nozzle at the bottom of the container was measured. The pourability measurements were performed immediately after preparing the suspensions and 48 h after previous mixing.

#### 2.4.4. Density Test

Density was measured at 20 °C using an aerometer. The suspension fertilizer was poured into the measuring cylinder immediately after its production, and the aerometer was placed in it in such a way that it would not touch the cylinder walls. After the aerometer level in the slurry had stabilized, the measurement result was read and recorded.

## 3. Results and Discussion

### 3.1. Selection of the Composition of Fertilizers

The suspension fertilizers presented in the study were dedicated to the cultivation of maize for silage due to its great importance in the production of food and feed. In later studies, it is planned to evaluate their effectiveness in field experiments with this plant.

The composition of the fertilizers was developed on the basis of recommendations for the cultivation of maize for green matter developed by the Institute of Soil Science and Plant Cultivation—National Research Institute in Puławy (IUNG) [21]. Maize extracts large amounts of nutrients and water from the soil. With a yield of 10 tons of forage, it consumes, on average: 38 kg of nitrogen (N), 16 kg of phosphorus (P_2_O_5_), 45 kg of potassium (K_2_O), 20 kg of calcium (CaO), 12 kg of magnesium (MgO), 5 kg of sulfur (S) or converted to SO_3_—12.5 kg and 17 g of boron (B), 13 g of copper (Cu), 150 g of manganese (Mn), 1.5 g of molybdenum (Mo), and 150 g of zinc (Zn). It shows high sensitivity to zinc deficiency and average sensitivity to boron, manganese, and copper deficiency. On this basis, the ratio of the main nutrients N, P, and K was established: 38:16:45 = 2.4:1:2.8.

For fertilizers with the addition of micronutrients, three micronutrients of key importance in maize cultivation were selected: boron, manganese, and zinc. The amount of these ingredients in suspension fertilizers was selected as the minimum amount that can be declared in a fertilizer containing primary and/or secondary nutrients with the addition of microelements for soil application in field crops according to the Regulation of the Minister of Economy on 8 September 2010 concerning the method of packaging mineral fertilizers, placing information about fertilizing ingredients on these packages. The method of testing mineral fertilizers and types of fertilizing lime amounts to: Zn—0.01% (m/m), Mn—0.1% (m/m), and B—0.01% (m/m). According to the new Regulation (EU) 2019/1009 of the European Parliament and of the Council [22], repealing Regulation (EC) No 2003/2003, which applies from 16 July 2022, manganese and zinc in liquid inorganic fertilizers can be declared with lower contents: Mn—0.01% (m/m) and Zn—0.002% (m/m). The new EU regulations do not pose a threat to the necessity to change in the adopted compositions for suspension fertilizers.

### 3.2. Assessment of the Suitability of Bentonites for the Stabilization of Fertilizer Suspensions

In order to determine the suitability of certain types of bentonites for the stabilization of fertilizer suspensions, their 12% aqueous solutions were prepared. After approximately 30 min, their swelling was assessed visually.

**Figure 1:**
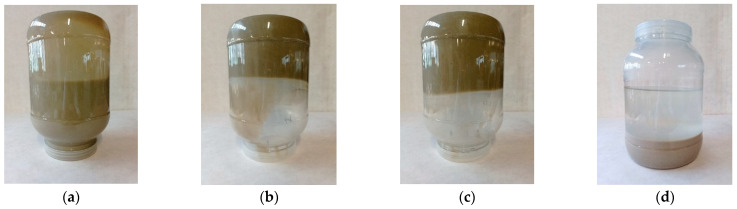
12% water solution of bentonite: (**a**) GM, (**b**) Special, (**c**) Special 45, and (**d**) SN 30 min after production.

The greatest swelling was observed in the solutions of Specjal and Specjal 45 bentonites. They took the form of a jelly-like substance that did not change position even when the container was turned upside down (Figure 1b,c). The GM bentonite formed a stable and fluid slurry (Figure 1a). However, the SN bentonite solution did not swell at all. After 30 min, it delaminated into a water layer at the top and a bentonite sediment at the bottom (Figure 1d). Therefore, it was not used in further studies aimed at stabilizing suspension fertilizers.

### 3.3. Assessment of Physical Parameters of Suspension Fertilizer Trials

#### 3.3.1. Assessment of the Density and Fluidity of Suspension Fertilizers

The proposed tests of suspension fertilizers without the addition of bentonite were prepared: tests 1.0–6.0 and with the addition of 3% of selected types of bentonites in the form of a 12% solution; with GM bentonite, tests 1.1–6.1; with Special 45 bentonite, tests 1.2–6.1; and with bentonite, Special tests 1.3–6.3. Table 9 shows the results of the density and fluidity tests for the given fertilizer samples. The test was carried out immediately after production and 48 h after mixing the bottle with the fertilizer by shaking it.

The density in fertilizers without the addition of bentonite did not change significantly after 48 h. Moreover, in fertilizers without bentonite with a higher concentration (tests 3.0 and 6.0), thicker crystals were formed, which clogged the outlet nozzle of the Ford cup. 

In fertilizers with the addition of bentonite, compared to fertilizers without their addition, the fluidity of the fertilizers improved after 48 h. In these tests, the washout time from the Ford cup was shortened, which was outside the recommended range immediately after being produced in the tests with the higher concentration. This is related to the structure produced by bentonite over time, which separates the grains of the dispersed phase. For this reason, the addition of bentonite facilitates the re-mixing of the fertilizer. The samples without the addition of bentonite required a longer shaking time in order to agitate the sediment collected at the bottom.

#### 3.3.2. Stability of Suspension Fertilizers

The graphs show the stability of the produced suspension fertilizer tests, as measured by the amount of clear liquid layer at the top in relation to the elapse of time.

**Figure 2:**
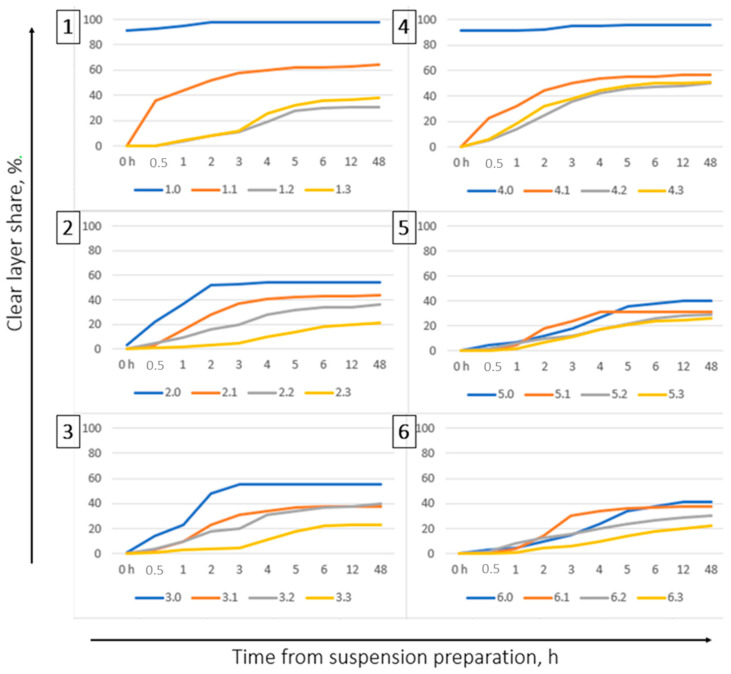
Graphs showing the stability of suspension fertilizer samples depending on the time that has elapsed since their production. Each graph concerns one type of fertilizer (1–6) in the variants of the addition of various bentonites.

In each case, the tests with the addition of bentonites had better stability than the tests without their addition. The best stability parameters are demonstrated by tests with Special bentonite, in which the smallest amount of clear liquid was produced (Figure 2 samples: 1.3–6.3). The greatest delamination was observed in the samples without bentonite, especially at those with a lower concentration (Figure 2 (1), (2) samples: 1.0–6.0). Moreover, the samples containing magnesium sulphate showed lower delamination: less than 60% for the samples 2 and 3 (Figure 2 (2), (3)) and less than 40% for the samples 5 and 6 (Figure 2 (5), (6), compared to the samples containing only the main nutrients (samples 1 and 4). The samples with the addition of bentonites were easily mixed (2–3 times by shaking the cilnder) and regained their initial stability.

#### 3.3.3. Viscosity of Suspension Fertilizers

Viscosity influences the suspension’s stability and its pourability. When the viscosity of the dispersion medium increases, the sink rate decreases, and the dispersed phase sinks slower and stays dispersed longer, resulting in a more stable suspension. However, as the viscosity of the system increases, the potential for leakage decreases, and a dosing problem arises. Highly viscous slurries consume energy excessively during the manufacturing process, so low viscosity is more cost-effective.

**Figure 3:**
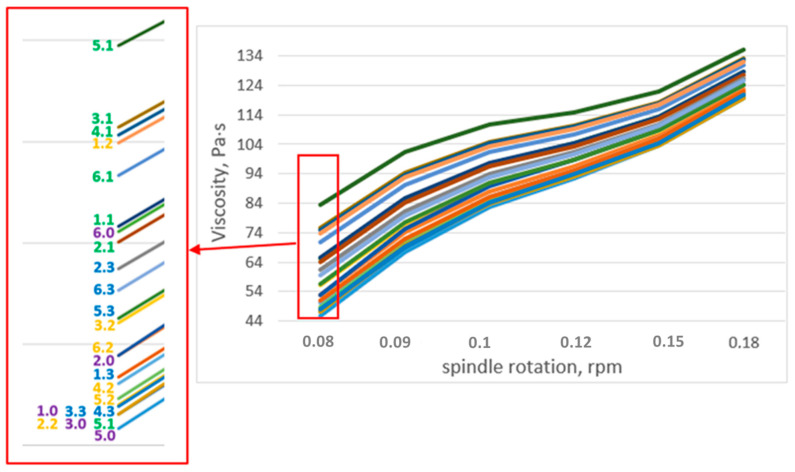
Dependence of the viscosity of fertilizer suspensions on the rotational speed of the measuring spindle.

The viscosity of all samples of suspension fertilizers increases with the increasing speed of rotation of the measuring spindle (Figure 3). This may be the result of solid particles falling to the bottom of the measuring container where the measuring spindle tip is located. This causes the suspension to thicken in this region and a greater resistance to the measuring spindle to occur. Overall, it was found that the tests with GM bentonite (1.1–6.1) had a higher viscosity than the other tests. On the other hand, the value of the viscosity of the samples without the addition of bentonite (1.0–6.0) is at the lowest level.

## 4. Conclusions

The presented research confirms the possibility of using waste phosphates from the production of polyols as raw materials for the production of fertilizers. Since this waste contains a large amount of water, suspension fertilizers are the most economically viable form of fertilizers produced. This form of fertilizer allows for a wide range of modification of the fertilizer composition. The waste raw material can be diluted with water or supplemented with appropriate raw materials that provide macro or micronutrient nutrients, depending on the needs. The solubility of the ingredients is not a limitation here. An important issue is the proper stabilization of the suspension with a stabilizing agent.

The suitability of bentonites for the stabilization of suspension fertilizers should be checked by prior preparation of the aqueous suspension and assessment of the degree of swelling. If bentonite does not form a stable suspension and delaminates, it is not suitable for preparation of suspension fertilizers on its basis. Addition of bentonites to fertilizer suspensions improves their rheological parameters, especially stability and fluidity. In the samples of fertilizers with the addition of bentonite, the fluidity improves with the passage of time.

The addition of bentonites to fertilizer suspensions prevents the formation of large crystals that could clog the nozzles during fertilizer application.

The addition of bentonite facilitates the restoration of the proper parameters of fertilizer suspensions after their remixing. All the proposed tests of fertilizers with the addition of bentonites, with a composition adapted to the nutritional requirements of maize, met the application criteria for suspension fertilizers.

In samples of high concentration, thicker crystals may develop during storage. This can cause clogging of the outlet nozzles during fertilizer application. This can result in an uneven distribution of the fertilizer in the field or a complete avoidance of its application.

In order to verify the agricultural efficiency, the proposed fertilizers are currently being tested in field experiments with maize intended for silage.

## Figures and Tables

**Table 1:** Urea ammonium nitrate solution (RSM).

Parameter	Unit	Value
Total N content	% wt.	2
Content NH_4_NO_3_	% wt.	44.3
Urea content	% wt.	35.4
Specific density	kg · m^−3^	1320
Crystallization temperature	°C	0

**Table 2:** Potassium salt—technical potassium chloride.

Parameter	Unit	Value
Content K_2_O	% wt.	60
Particle content less than 0.6 mm	% wt.	min. 80
Angle of repose	degree	35
Bulk density	kg · m^−3^	1110

**Table 3:** Magnesium sulfate 7 hydrate.

Parameter	Unit	Value
Content MgO	%	16
Content SO_3_	%	32

**Table 4:** Boric acid.

Parameter	Unit	Value
Type		p.a.
Content	%	min. 99.5%

**Table 5:** Zinc Sulphate 7 hydrate.

Parameter	Unit	Value
Type		p.a.
Content	%	min. 99.5%
pH (5%, H_2_O)		4.4–5.6

**Table 6:** Manganu (II) siracha 1 hydrate.

Parameter	Unit	Value
Type		p.a.
Content	%	min. 99.5%

**Table 7:** Composition of fertilizers of the 1st series with the main composition of NPK: 9.5–4-11.

Sample	Type of Fertilizer	The Content of Fertilizing Ingredients, %							
		N	P_2_O_5_	K_2_O	MgO	S	Zn	Mn	B
1	NPK	9.5	**4**	11	-	-	-	-	-
2	NPK + MgO + S	9.5	**4**	11	3	6	-	-	-
3	NPK + MgO + S + microelements	9.5	**4**	11	3	6	0.01	0.1	0.01

**Table 8:** Composition of fertilizers of the 1st series with the main composition of NPK: 9.5–6-11.

Sample	Type of Fertilizer	The Content of Fertilizing Ingredients, %							
		N	P_2_O_5_	K_2_O	MgO	S	Zn	Mn	B
4	NPK	9.5	**6**	11	-	-	-	-	-
5	NPK + MgO + S	9.5	**6**	11	3	6	-	-	-
6	NPK + MgO + S + microelements	9.5	**6**	11	3	6	0.01	0.1	0.01

**Table 9:** Physical parameters of the produced samples of suspension fertilizers.

		Density, g/cm^3^		Castability, s	
	Recommended Range	1200–1400		10–15	
Bentonite	Sample	After 0 h	After 48 h	After 0 h	After 48 h
-	1.0	1.251	1.260	10.35	9.70
	2.0	1.378	1.372	11.24	10.54
	3.0	1.378	1.373	11.91	*
	4.0	1.292	1.285	10.22	9.76
	5.0	1.435	1.406	13.46	10.87
	6.0	1.546	1.414	18.71	*
GM	1.1	1.262	1.262	10.23	9.74
	2.1	1.251	1.350	16.64	10.82
	3.1	1.250	1.344	15.60	10.92
	4.1	1.287	1.282	10.53	9.76
	5.1	1.454	1.381	34.09	11.71
	6.1	1.482	1.390	22.92	10.76
Specjal 45	1.2	1.267	1.272	9.98	10.01
	2.2	1.235	1.343	16.15	10.72
	3.2	1.297	1.337	17.83	10.63
	4.2	1.285	1.286	10.43	9.87
	5.2	1.368	1.375	20.36	10.72
	6.2	1.565	1.364	38.24	11.20
Specjal	1.3	1.263	1.270	9.72	9.78
	2.3	1.227	1.269	20.72	11.75
	3.3	1.260	1.281	21.16	12.38
	4.3	1.275	1.291	10.11	10.44
	5.3	1.447	1.346	30.58	12.88
	6.3	1.710	1.367	39.43	11.64

* clogging of the nozzle by large, rapidly falling crystals.

## Data Availability

The data presented in this study are available on request from the corresponding author.

## Sample Availability

Samples of the compounds are not available from the authors.
